# Steatotic liver disease indices for cardiovascular event prediction: Panasonic cohort study 28

**DOI:** 10.1016/j.ajpc.2026.101541

**Published:** 2026-03-10

**Authors:** Nozomi Kuramoto, Hiroshi Okada, Hanako Nakajima, Naoko Nakanishi, Emi Ushigome, Masahide Hamaguchi, Toshiaki Ohkuma, Daisuke Tanaka, Teruki Miyake, Kazushiro Kurogi, Hiroaki Murata, Eri Tsuda, Naoki Yoshida, Masato Ito, Michiaki Fukui

**Affiliations:** a Department of Endocrinology and Metabolism, Graduate School of Medical Science, Kyoto Prefectural University of Medicine, 465 Kajii-cho, Kawaramachi-Hirokoji, Kamigyo-ku, Kyoto, Japan; b Department of Medicine and Clinical Science, Graduate School of Medical Sciences, Kyushu University, Fukuoka, Japan; c Department of Diabetes and Endocrinology, Shiga General Hospital; d Department of Lifestyle-Related Medicine and Endocrinology, Ehime University Graduate School of Medicine, Matsuyama, Shikoku, Japan; e Department of Health Care Center, Panasonic Health Insurance Organization, Moriguchi, Japan.; f Department of Orthopaedic Surgery, Matsushita Memorial Hospital, Moriguchi, Japan.

**Keywords:** Steatotic liver disease, Cardiovascular disease, Fatty liver index, Risk prediction, Population-based cohort study, Japanese population, Hepatic steatosis indices

## Abstract

**Background:**

Noninvasive hepatic steatosis indices may aid in cardiovascular risk assessment; however, their comparative predictive abilities remain uncertain.

**Methods:**

We analyzed data from 134,604 Japanese adults aged ≥ 40 years who underwent health checkups between 2011 and 2020. Four indices (the ZJU index, Hepatic Steatosis Index (HSI), Fatty Liver Index (FLI), and K-NAFLD score) were evaluated for their ability to predict major adverse cardiovascular events (MACE). Time-dependent receiver operating characteristic (ROC) analyses based on 10-year follow-up were performed using univariable Cox models including each index. The area under the ROC curve (AUC) was calculated to assess predictive performance. We compared AUCs between pairs of indices to determine which performed better, using 1,000 bootstrap resamples and 95 % confidence intervals.

**Results:**

During a total follow-up period of approximately 860,000 person-years, 3,532 participants developed MACE (incidence: 4 per 1,000 person-years). All indices were significantly associated with MACE incidence. FLI showed the highest AUC for predicting MACE (0.615), followed by the K-NAFLD score (0.613), with optimal cutoffs of 23.33 and –2.61, respectively. Both indices outperformed the ZJU index and HSI, with statistically significant differences in AUCs. No significant difference was observed between FLI and the K-NAFLD score.

**Conclusions:**

The FLI and K-NAFLD score demonstrated relatively better predictive performance for cardiovascular events compared with the ZJU index and HSI. These findings may support their use in public health screening and clinical risk stratification.

## Graphical abstract

**Figure:**
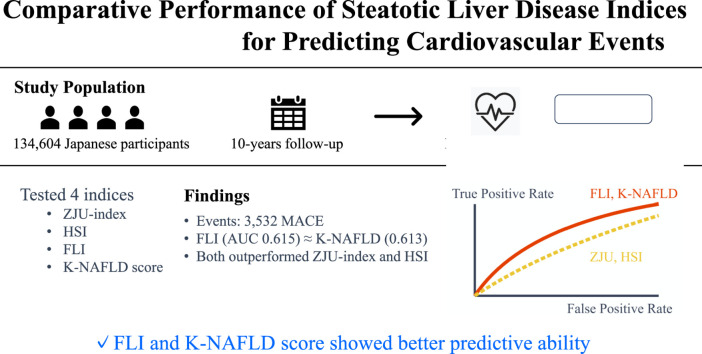

## 1 Introduction

Steatotic liver disease (SLD), the prevalence of which has been increasing in recent years, is now one of the most common chronic liver diseases worldwide [[1], [2], [3], [4]] SLD is not solely a liver-specific condition but is closely associated with metabolic disorders such as type 2 diabetes, metabolic syndrome, and cardiovascular disease (CVD) [3,[5], [6], [7]] Given that patients with SLD have higher all-cause and cardiovascular mortality rates, [[8], [9], [10], [11]] early diagnosis and accurate risk assessment are essential.

Although abdominal ultrasonography is commonly used to diagnose SLD, it is impractical for large-scale screening or routine health checkups. Alternatively, various hepatic steatosis indices have been proposed to estimate SLD based on blood test results and anthropometric measurements. Representative examples include the ZJU index, [12] Hepatic Steatosis Index (HSI), [13] Fatty Liver Index (FLI), [14] and K-NAFLD score [15]

Hepatic steatosis has been increasingly recognized as a metabolic disorder with systemic implications, particularly for cardiovascular health. Multiple cohort studies have reported that individuals with metabolic dysfunction–associated steatotic liver disease (MASLD; previously termed non-alcoholic fatty liver disease [NAFLD]) or related hepatic phenotypes have a significantly elevated risk of cardiovascular events, independent of conventional risk factors such as hypertension, dyslipidemia, and diabetes.

In this context, noninvasive hepatic steatosis indices may serve not only as tools for identifying liver fat accumulation but also as simple and cost-effective markers for cardiovascular risk stratification. Several of these indices, including FLI, HSI, and K-NAFLD score, have been associated with cardiovascular events [[16], [17], [18], [19], [20]] However, few studies have compared the predictive abilities of these indices related to cardiovascular events, and the relative performance of each index in predicting CVD risk remains unclear.

Therefore, the aim of this study was to compare the predictive abilities of four hepatic steatosis indices (the ZJU index, HSI, FLI, and K-NAFLD score) for cardiovascular events in a large-scale, long-term Japanese cohort study.

## 2 Methods

### 2.1 Study design and data collection

This study utilized data from the Panasonic Cohort Study, which includes participants from a health screening program conducted by the Panasonic Corporation (Osaka, Japan) across 166 operational sites. The program was designed to promote employee health by detecting chronic conditions such as impaired glucose tolerance, dyslipidemia, and hypertension at an early stage, and by assessing modifiable lifestyle risk factors such as diet and physical activity. The cohort database consists of data from annual health checkups, self-administered questionnaires, and mortality records. All Panasonic employees underwent annual health examinations, including anthropometric measurements and blood and urine tests. Body weight and height were measured with participants wearing light indoor clothing and no shoes, and body mass index (BMI) was calculated as weight (kg) divided by height squared (m²). Waist circumference was measured at the level of the umbilicus in a standing position. Blood pressure was measured in the seated position using an automated sphygmomanometer after a brief rest period, and both systolic and diastolic blood pressures were recorded. Blood samples were collected after a minimum of 10 hours of fasting, and routine laboratory assays were used to measure fasting plasma glucose, triglycerides, liver enzymes, and lipid profiles.

The self-administered questionnaire included items on smoking status, physical activity, and treatment for metabolic disorders. Smoking status was categorized as never, former, or current smoker. “Non/low alcohol intake” was defined as <20 g/day for women and <30 g/day for men. Participants who engaged in regular physical activity—defined as exercising for ≥30 minutes per session, at least twice per week, for more than one year—were categorized as regular exercisers. Further details of this cohort study have been described in a previous publication [21]

This study included individuals aged ≥40 years who underwent health checkups between 2011 and 2020. The first health checkup within the observation period was defined as the baseline. Participants were excluded if they had missing baseline data, a history of CVD at baseline, or only one health checkup record during the study period.

This cohort study was approved by the Regional Ethics Committee of the Panasonic Health Insurance Association (Approval Number: 2021-001) and was conducted in accordance with the principles of the Declaration of Helsinki.

The datasets used in this study are available from the corresponding author upon reasonable request.

### 2.2 Cardiovascular events definition

Major adverse cardiovascular events (MACE) were defined according to commonly used standard definitions as a composite of non-fatal myocardial infarction, non-fatal stroke, and cardiovascular death. Mortality data were extracted from the database, whereas information on coronary artery disease (CAD) and stroke was obtained from standardized annual self-administered questionnaires used in the Japanese Specific Health Checkup system. CAD was defined as angina pectoris or myocardial infarction, and stroke was defined as cerebral infarction or cerebral hemorrhage, based on physician diagnosis or treatment history reported by participants. Independent blinded adjudication of cardiovascular events was not available in this occupational cohort; however, outcome information was obtained through self-administered questionnaires within a standardized health checkup system, and the health checkup data were collected and managed by personnel independent of the present study.

### 2.3 Hepatic steatosis indices calculation

The following formulas were used to calculate each hepatic steatosis index:

ZJU index = body mass index (BMI, kg/m²) + fasting plasma glucose (FPG, mmol/L) + triglycerides (TG, mmol/L) + 3 × (alanine aminotransferase/aspartate aminotransferase [ALT/AST] ratio) (+2 if female) [12]

HSI = 8 × (ALT/AST ratio) + BMI (+2 if diabetic, +2 if female) [13]

$$FLI\;=\;\frac{e^{0.953*\ln\left(TG\right)+0.139*BMI+0.718*\ln\left(GGT\right)+0.053*WC-15.745}}{1+e^{0.953*\ln\left(TG\right)+0.139*BMI+0.718*\ln\left(GGT\right)+0.053*WC-15.745}}*100\;\left[14\right]$$

K-NAFLD score = 0.913 × (2 if female; 1 if male) + 0.089 × waist circumference (WC) + 0.032 × (systolic blood pressure + FPG) + 0.007 × TG + 0.105 × ALT − 20.929 [15]

The individual components included in each hepatic steatosis index are summarized in Supplemental Table 1.

### 2.4 Statistical analysis

Statistical analyses were performed using JMP Pro, version 18.1.1 (SAS Institute Inc., Cary, NC, USA) and R (The R Foundation for Statistical Computing, Vienna, Austria). A *p*-value of < 0.05 was considered statistically significant.

The follow-up period was defined as the time from baseline to the onset of the outcome, the end of consecutive health examinations, or the end of the cohort study, whichever occurred first.

Baseline characteristics were presented as medians with interquartile ranges, or as counts and percentages, as appropriate.

The primary outcome was the incidence of MACE. Secondary outcomes included the incidence of CAD and stroke.

First, associations between each hepatic steatosis index and cardiovascular events were evaluated using multivariable Cox proportional hazard models. The models were adjusted for age, smoking status, alcohol consumption, physical activity, HDL cholesterol, LDL cholesterol, and use of diabetes, antihypertensive, and lipid-lowering medications. As sensitivity analyses, we additionally fitted models including sex as a covariate. As the FLI, K-NAFLD score, and HSI were not normally distributed, they were log-transformed before analysis. Associations were expressed as hazard ratios (HRs) with 95 % confidence intervals (CIs). To assess whether the addition of each steatotic liver disease index improved the predictive ability for cardiovascular events, likelihood ratio tests were conducted by comparing the base model with and without each index.

To assess the predictive performance of each index for cardiovascular events, time-dependent receiver operating characteristic (ROC) analyses were conducted based on a 10-year follow-up, using univariable Cox models that included each hepatic steatosis index individually. The area under the ROC curve (AUC) was calculated using the survival ROC package in R. Optimal cutoff values were determined using the Youden index, and the corresponding sensitivity, specificity, positive predictive value, negative predictive value, positive likelihood ratio, and negative likelihood ratio were also calculated.

Differences in AUCs and their 95 % CIs were calculated to compare the predictive abilities of the hepatic steatosis indices. Statistical significance between indices was assessed using the bootstrap method with 1,000 resamples, implemented via the boot package in R. For each pairwise comparison, the difference in AUC, corresponding 95 % CI, and *p*-value were reported.

To evaluate clinical applicability, additional analyses were conducted by fixing specificity at 70 %, 80 %, and 90 %, and calculating the predictive performance of each hepatic steatosis index under these conditions. These analyses aimed to assess the contribution of each index to the prediction of cardiovascular events (MACE, CAD, and stroke) across a range of clinically relevant thresholds, reflecting different balances between sensitivity and specificity.

For model performance evaluation, we constructed a base model including age, sex, body mass index, smoking status, physical activity, alcohol intake, HDL cholesterol, LDL cholesterol, and medication use for hypertension, dyslipidemia, and diabetes. Each hepatic steatosis index was then added individually to this base model. Incremental model performance was assessed using likelihood ratio tests, calibration plots based on grouped 10-year Kaplan–Meier estimates, and reclassification metrics including integrated discrimination improvement (IDI) and continuous net reclassification improvement (cNRI). For calibration analyses, participants were divided into 20 groups according to predicted risk, and the mean predicted risk in each group was compared with the observed 10-year event probability estimated using Kaplan–Meier methods.

Furthermore, a subgroup analysis was performed among participants aged ≥50 years. The cutoff age of 50 years was selected considering age-related changes in cardiovascular risk profiles and its use in previous studies on hepatic steatosis and cardiometabolic outcomes [22,23]

In an additional exploratory analysis, participants were stratified according to cardiovascular risk status. A high-risk group was defined as individuals presenting with two or more of the following cardiometabolic risk factors: impaired glucose regulation, hypertension, dyslipidemia, current smoking, or body mass index (BMI) ≥25 kg/m². Impaired glucose regulation was defined as fasting plasma glucose ≥100 mg/dL or the use of antidiabetic medication. Hypertension was defined as systolic blood pressure ≥130 mmHg, diastolic blood pressure ≥85 mmHg, or the use of antihypertensive medication. Dyslipidemia was defined as triglycerides ≥150 mg/dL, LDL cholesterol ≥140 mg/dL, HDL cholesterol <40 mg/dL in men or <50 mg/dL in women, or the use of lipid-lowering medication.

## 3 Results

**Figure 1:**
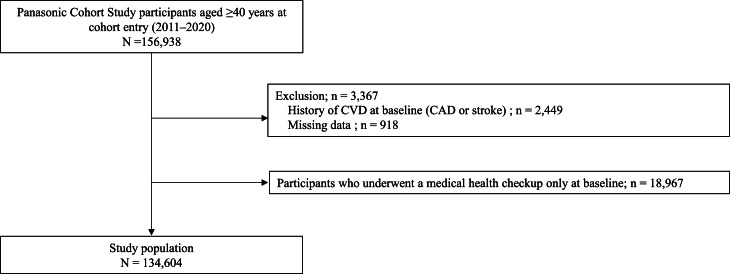
**Flowchart of participant selection for the present study.** This study included individuals aged 40 years or older who underwent health checkups between 2011 and 2020. The first health checkup during this period was defined as baseline. Participants were excluded if they had a history of cardiovascular disease at baseline, missing baseline data on key variables, or no follow-up health checkup after baseline.

The selection process for study participants is summarized in Fig. 1. A total of 156,938 individuals aged 40 years or older who underwent health checkups between 2011 and 2020 were initially considered for inclusion. Of these, 22,334 individuals were excluded for the following reasons: 18,967 did not undergo any health checkup after baseline; 2,449 had a history of CVD at baseline; and 918 had missing data on key variables, including height, weight, WC, blood pressure, laboratory measurements, use of diabetes, antihypertensive, or lipid-lowering medications, smoking status, physical activity, and alcohol consumption. Thus, 134,604 participants were included in the final analysis.

Baseline characteristics of the participants are presented in Table 1. The median age was 45 years (interquartile range 40–50), 59.5 % were men, and the median body mass index was 22.9 kg/m². The total observation period comprised approximately 860,000 person-years. During the study period, 3,532 participants developed MACE, corresponding to an incidence rate of approximately 4 cases per 1,000 person-years. Among 3,532 participants who developed MACE, the first event was coronary artery disease (CAD) in 2,290 (64.8 %), stroke in 1,122 (31.8 %), and cardiovascular death in 120 (3.4 %).

**Table 1:** Characteristics of participants at baseline.

N	134604
Age(y)	45(40-50)
Men, n ( %)	102227 (75.9 %)
BMI(kg/m²)	22.9(20.9-25.2)
Height(cm)	169.0(163.1-173.7)
WC(cm)	82.0(75.9-88.0)
ZJU-index	32.8(30.1-36.1)
HSI	31.3(28.3-35.5)
FLI	19.6(7.3-44.2)
K-NAFLD score	-2.8(-4.2–0.9)
Systolic blood pressure(mmHg)	119(109-128)
Diastolic blood pressure(mmHg)	74(67-82)
Fasting plasma glucose(mg/dL)	92.0(87.0-99.0)
Triglycerides(mg/dL)	91.0(63.0-136.0)
HDL cholesterol(mg/dL)	58.0(49.0-69.0)
LDL cholesterol(mg/dL)	123.0(103.0-145.0)
AST(IU/L)	23.0(17.0-25.0)
ALT(IU/L)	20.0(14.0-30.0)
GGT(IU/L)	28.0(19.0-48.0)
smoking(none/past/current),n,( %)	70205/24247/40150 (52.2/18.0/30.0)
Alcohol consumption,n,(+)( %)	28116 (20.1)
Physical exercise,n,(+)( %)	24655 (18.3)
Diabetes medication use, n, (+)( %)	3239(2.4)
Antihypertensive medication use, n, (+)( %)	10925(8.0)
Dyslipidemia medication use, n, (+)( %)	5513(4.1)

For categorical variables, n ( %) is presented. For continuous variables, median (interquartile range) is presented. Alcohol consumption (+) indicates alcohol intake above the predefined threshold (≥20 g/day for women and ≥30 g/day for men).

Abbreviations: BMI, body mass index; WC, waist circumference; ZJU index, Zhejiang University Index; HSI, Hepatic Steatosis Index; FLI, Fatty Liver Index; K-NAFLD score, Korean National Health and Nutrition Examination Survey Non-Alcoholic Fatty Liver Disease Score; LDL, low-density lipoprotein; HDL, high-density lipoprotein.

As shown in Table 2, all hepatic steatosis indices (ZJU index, log-transformed HSI, log-transformed FLI, and log-transformed K-NAFLD score) were significantly associated with the incidence of cardiovascular events after adjustment for confounding factors. Although the magnitude of the HRs varied among the indices, they ranged from 1.03 to 1.26 across all outcomes. The associations remained materially similar in sensitivity analyses additionally adjusting for sex (Supplemental Table 2).

**Table 2:** Multivariable-adjusted hazard ratios for cardiovascular disease.

MACE	aHR	95 % CI
ZJU index (per SD)	1.07	(1.02–1.08)
logHSI (per SD)	1.08	(1.04–1.12)
logFLI (per SD)	1.23	(1.18–1.30)
logK-NAFLD score (per SD)	1.19	(1.14–1.23)
CAD	aHR	95 % CI
ZJU index (per SD)	1.03	(1.02–1.04)
logHSI (per SD)	1.09	(1.04–1.14)
logFLI (per SD)	1.26	(1.18–1.33)
logK-NAFLD score (per SD)	1.21	(1.16–1.27)
Stroke	aHR	95 % CI
ZJU index (per SD)	1.20	(1.03–1.40)
logHSI (per SD)	1.07	(1.00–1.15)
logFLI (per SD)	1.18	(1.09–1.28)
logK-NAFLD score (per SD)	1.15	(1.07–1.23)

The multivariate model was adjusted for age, smoking status, alcohol consumption, physical activity, HDL cholesterol, LDL cholesterol, use of diabetes medication, use of antihypertensive medication, and use of dyslipidemia medication.

Abbreviations: MACE, major adverse cardiovascular event; CAD, coronary artery disease; aHR, adjusted hazard ratio; CI, confidence interval; ZJU index, Zhejiang University Index; HSI, Hepatic Steatosis Index; FLI, Fatty Liver Index; K-NAFLD score, Korean National Health and Nutrition Examination Survey Non-Alcoholic Fatty Liver Disease Score.

In likelihood ratio tests, the addition of each steatotic liver disease index to the base model significantly improved the prediction of all cardiovascular events (all *p* < 0.05; Supplemental Table 3).

**Figure 2:**
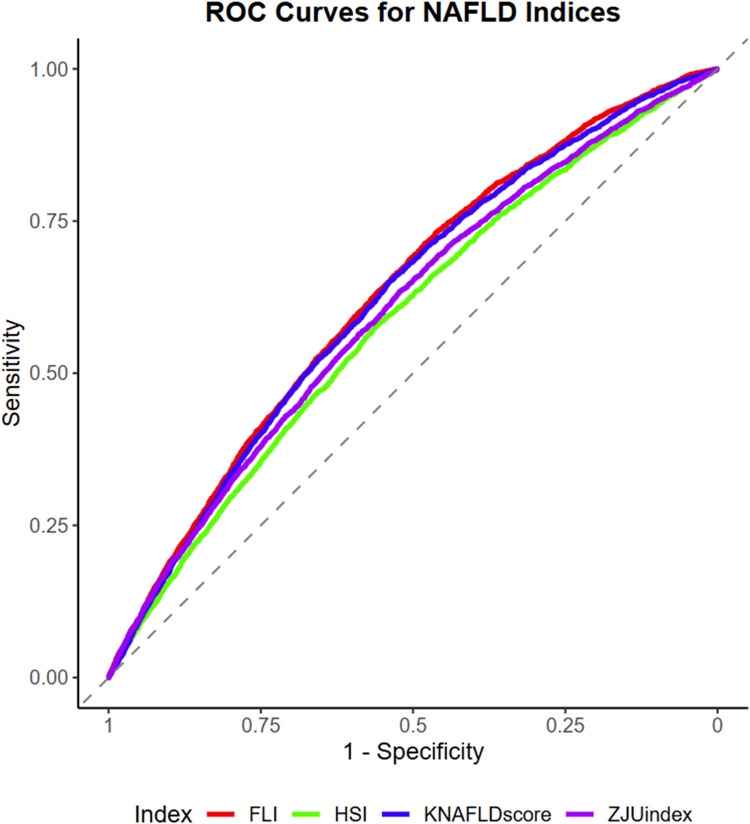
**ROC curves for predicting cardiovascular events in participants aged ≥40 years.** Time-dependent receiver operating characteristic (ROC) curves for predicting major adverse cardiovascular events over 10 years using four hepatic steatosis indices: Zhejiang University (ZJU) index, Hepatic Steatosis Index (HSI), Fatty Liver Index (FLI), and Korean Non-alcoholic Fatty Liver Disease (K-NAFLD) score. Analyses were performed in participants aged 40 years and older. Each curve was derived from a univariable Cox proportional hazards model including one index, and the area under the curve (AUC) was calculated for each.

Table 3 presents the 10-year AUC for each cardiovascular event. For MACE prediction, the FLI showed the highest AUC (0.615), closely followed by the K-NAFLD score (0.613), whereas the ZJU index and HSI demonstrated lower discrimination (AUCs 0.594 and 0.575, respectively). The corresponding optimal cutoff values, as well as the sensitivity and specificity at those cutoffs, are also reported. Fig. 2 illustrates the results for MACE.

**Table 3:** Area under the curve and optimal cut-offs for cardiovascular outcomes.

MACE								
Model	AUC of ROC at 10 years (95 % CI)	Cut-off value (95 % CI)	Sensitivity (95 % CI)	Specificity (95 % CI)	NPV (95 % CI)	PPV (95 % CI)	NLR (95 % CI)	PLR (95 % CI)
ZJU-index	0.59 (0.58–0.60)	32.94 (32.0–34.2)	61.8 % (50.8–70.0)	52.4 % (44.1–63.0)	96.8 % (96.5–97.0)	5.6 % (5.4–6.1)	0.73 (0.68–0.79)	1.30 (1.25–1.40)
HSI	0.58 (0.57–0.59)	32.12 (30.9–32.8)	55.8 % (50.3–64.5)	56.1 % (47.4–61.0)	96.5 % (96.3–96.7)	5.5 % (5.2–5.8)	0.79 (0.75–0.82)	1.27 (1.22–1.32)
FLI	0.62 (0.61–0.62)	23.33 (16.0–32.0)	61.2 (50.9–71.8)	55.8 % (45.7–66.0)	96.9 % (96.7–97.3)	6.0 % (5.5–6.5)	0.70 (0.62–0.75)	1.39 (1.30–1.52)
K-NAFLD score	0.61 (0.60–0.62)	-2.61 (-3.10–-1.80)	63.3 % (49.9–71.0)	54.1 % (46.5–66.7)	97.0 % (96.6–97.3)	5.9 % (5.6–6.6)	0.68 (0.62–0.75)	1.38 (1.31–1.53)
CAD								
Model	AUC of ROC at 10 years (95 % CI)	Cut-off value (95 % CI)	Sensitivity (95 % CI)	Specificity (95 % CI)	NPV (95 % CI)	PPV (95 % CI)	NLR (95 % CI)	PLR (95 % CI)
ZJU-index	0.60 (0.59–0.61)	33.97 (32.01–34.53)	54.3 % (49.2–70.4)	60.8 % (44.0–65.1)	97.7 % (97.6–98.0)	4.2 % (3.8–4.5)	0.75 (0.66–0.78)	1.38 (1.26–1.47)
HSI	0.58 (0.57–0.59)	32.12 (31.46–34.48)	56.9 % (42.1–60.7)	56.0 % (51.9–70.8)	97.6 % (97.4–97.8)	3.9 % (3.7–4.4)	0.77 (0.73–0.83)	1.29 (1.24–1.44)
FLI	0.62 (0.61–0.63)	23.33 (17.01–31.98)	62.7 % (52.1–70.3)	55.6 % (47.3–65.8)	97.9 % (97.7–98.1)	4.3 % (4.0–4.7)	0.67 (0.62–0.73)	1.41 (1.34–1.54)
K-NAFLD score	0.62 (0.61–0.63)	-2.61 (-3.01–-1.72)	64.0 % (49.0–70.8)	53.9 % (48.0–68.0)	97.9 % (97.6–98.1)	4.2 % (4.0–4.9)	0.67 (0.61–0.75)	1.39 (1.32–1.61)
Stroke								
Model	AUC of ROC at 10 years (95 % CI)	Cut-off value (95 % CI)	Sensitivity (95 % CI)	Specificity (95 % CI)	NPV (95 % CI)	PPV (95 % CI)	NLR (95 % CI)	PLR (95 % CI)
ZJU-index	0.58 (0.57–0.60)	32.94 (31.89–35.41)	60.9 % (42.1–70.3)	51.9 % (42.7–70.8)	98.9 % (98.8–99.0)	1.8 % (1.6–2.0)	0.75 (0.69–0.83)	1.27 (1.20–1.42)
HSI	0.57 (0.55–0.58)	32.38 (29.01–33.63)	52.4 % (44.3–77.8)	57.5 % (31.7–65.4)	98.8 % (98.7–99.1)	1.7 % (1.5–1.9)	0.83 (0.66–0.86)	1.23 (1.13–1.32)
FLI	0.58 (0.55–0.61)	16.78 (10.98–32.02)	69.6 % (49.1–78.3)	45.9 % (36.1–65.5)	99.1 % (98.9–99.2)	1.8 % (1.7–2.1)	0.66 (0.58–0.78)	1.29 (1.23–1.49)
K-NAFLD score	0.58 (0.55–0.61)	-2.67 (-3.51–-2.49)	63.1 % (58.5–77.2)	52.6 % (38.8–56.4)	99.0 % (98.9–99.2)	1.9 % (1.7–2.0)	0.70 (0.58–0.75)	1.33 (1.23–1.41)

Time-dependent receiver operating characteristic (ROC) analyses were conducted using univariable Cox models including each hepatic steatosis index. The area under the ROC curve (AUC) and corresponding 95 % CIs were calculated for 10-year follow-up. Optimal cut-off values were determined using the Youden index, with corresponding sensitivity, specificity, negative predictive value (NPV), positive predictive value (PPV), negative likelihood ratio (NLR), and positive likelihood ratio (PLR). Abbreviations: MACE, major adverse cardiovascular events; CAD, coronary artery disease; AUC, area under the ROC curve; NPV, negative predictive value; PPV, positive predictive value; NLR, negative likelihood ratio; PLR, positive likelihood ratio; ZJU index, Zhejiang University Index; HSI, Hepatic Steatosis Index; FLI, Fatty Liver Index; K-NAFLD score, Korean National Health and Nutrition Examination Survey Non-Alcoholic Fatty Liver Disease Score.

For the prediction of MACE, the FLI demonstrated the highest AUC, followed by the K-NAFLD score. The optimal cutoff values for these indices were 23.33 and –2.61, respectively.

Similarly, for CAD, the FLI and K-NAFLD score showed the highest AUCs among the indices.

In the prediction of stroke, both the FLI and K-NAFLD score demonstrated relatively higher AUCs than the ZJU index and HSI; however, the overall AUC values for stroke were slightly lower than those for MACE and CAD.

Table 4 shows the differences in AUCs among the hepatic steatosis indices for the prediction of MACE, CAD, and stroke. For all three outcomes, both the FLI and K-NAFLD score demonstrated significantly higher predictive ability than the ZJU index and HSI. However, there was no significant difference between the FLI and K-NAFLD score. Additionally, HSI demonstrated significantly better predictive ability than the ZJU index.

**Table 4:** Pairwise comparisons of AUCs for predicting cardiovascular outcomes.

MACE			
Comparison	ΔAUC	95 % CI	P value
HSI - ZJU index	-0.019	-0.023–0.015	<0.001
FLI - ZJU index	0.021	0.016–0.026	<0.001
K-NAFLD score - ZJU index	0.020	0.014–0.025	<0.001
FLI - HSI	0.040	0.034–0.046	<0.001
K-NAFLD score - HSI	0.038	0.032–0.044	<0.001
K-NAFLD score - FLI	-0.002	-0.006–0.003	0.58
CAD			
Comparison	ΔAUC	95 % CI	P value
HSI - ZJU index	-0.019	-0.024– -0.015	<0.001
FLI - ZJU index	0.013	0.013–0.025	<0.001
K-NAFLD score - ZJU index	0.013	0.013–0.025	<0.001
FLI - HSI	0.039	0.031–0.046	<0.001
K-NAFLD score - HSI	0.039	0.031–0.0455	<0.001
K-NAFLD score - FLI	-0.0002	-0.006–0.006	0.936
Stroke			
Comparison	ΔAUC	95 % CI	P value
HSI - ZJU index	-0.017	-0.022– -0.011	<0.001
FLI - ZJU index	0.018	0.009–0.026	<0.001
K-NAFLD score - ZJU index	0.017	0.001–0.025	<0.001
FLI - HSI	0.035	0.023–0.044	<0.001
K-NAFLD score - HSI	0.033	0.024–0.043	<0.001
K-NAFLD score - FLI	-0.001	-0.010–0.008	0.822

Pairwise differences in AUCs between hepatic steatosis indices were calculated using 1,000 bootstrap resamples to determine 95 % CIs and p-values. Abbreviations: MACE, major adverse cardiovascular events; CAD, coronary artery disease; AUC, area under the ROC curve; CI, confidence interval; ZJU index, Zhejiang University Index; HSI, Hepatic Steatosis Index; FLI, Fatty Liver Index; K-NAFLD score, Korean National Health and Nutrition Examination Survey Non-Alcoholic Fatty Liver Disease Score.

The predictive performance of each hepatic steatosis index under fixed specificity conditions (70 %, 80 %, and 90 %) are summarized in Supplemental Tables 4–6. At the predefined cutoff values, the K-NAFLD score and FLI showed the highest sensitivity for predicting MACE, CAD, and stroke. However, under stricter specificity conditions (80 % and 90 %), the ZJU index tended to retain higher sensitivity than the other indices.

Calibration was generally good across models, although calibration in the highest predicted-risk range may be less stable due to sparse data (Supplemental Figure 1). Reclassification analyses demonstrated significant improvement in risk stratification for FLI (cNRI 0.074, 95 % CI 0.036–0.113) and K-NAFLD score (cNRI 0.083, 95 % CI 0.046–0.121) compared with the base model, whereas ZJU index and HSI did not show significant reclassification improvement (Supplemental Table 7). IDI values were small for all indices, indicating limited incremental discrimination.

A subgroup analysis was conducted among participants aged ≥50 years. Baseline characteristics of this subgroup are presented in Supplemental Table 8.

**Figure 3:**
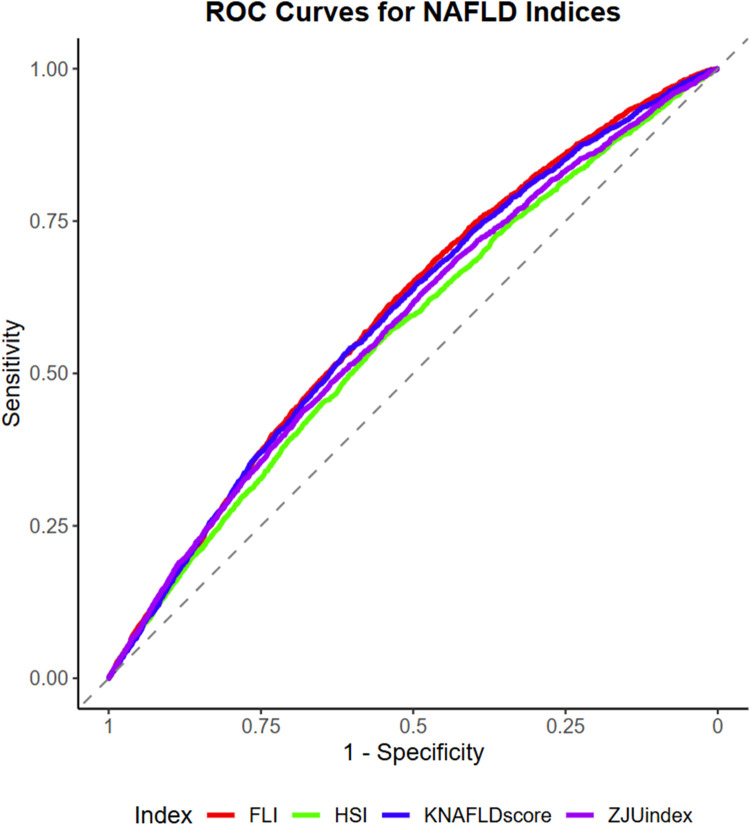
**ROC curves for predicting cardiovascular events in participants aged ≥50 years.** Time-dependent receiver operating characteristic (ROC) curves for predicting major adverse cardiovascular events over 10 years using four hepatic steatosis indices: Zhejiang University (ZJU) index, Hepatic Steatosis Index (HSI), Fatty Liver Index (FLI), and Korean Non-alcoholic Fatty Liver Disease (K-NAFLD) score. Analyses were performed in participants aged 50 years and older. Each curve was derived from a univariable Cox proportional hazards model including one index, and the area under the curve (AUC) was calculated for each.

When comparing the results of the overall analysis (participants aged ≥40 years) with those of the subgroup analysis (aged ≥50 years), similar trends were observed in the associations between hepatic steatosis indices and the incidence of cardiovascular events (Supplemental Tables 9–11). Fig. 3 illustrates the results of the subgroup analysis for MACE.

In an additional analysis restricted to participants with a higher cardiovascular risk profile (defined as having two or more of the following conditions: impaired glucose regulation, hypertension, dyslipidemia, current smoking, or BMI ≥25 kg/m²), the overall predictive performance of the hepatic steatosis indices tended to be lower than that observed in the main analysis. The results of this analysis are presented in Supplemental Table 12. The pairwise comparisons of AUC values among the hepatic steatosis indices within the high-risk group are presented in Supplementary Table 13. Overall, the relative differences between indices were broadly consistent with those observed in the main analysis. Although the relative ranking between FLI and the K-NAFLD score for CAD slightly differed from the main analysis, the difference in AUC between these two indices was small and not statistically significant.

## 4 Discussion

In this study, we compared the 10-year predictive abilities of four hepatic steatosis indices (the ZJU index, HSI, FLI, and K-NAFLD score) for cardiovascular events. Both the FLI and K-NAFLD score showed significantly higher predictive performance than the ZJU index and HSI.

Although the AUC values for individual indices were relatively low, this finding was not unexpected given the multifactorial nature of CVD. In fact, the AUCs were approximately 0.61, indicating modest discrimination and suggesting that these indices alone are unlikely to provide strong individual-level risk stratification. Moreover, because established cardiovascular risk scores incorporate a broader range of predictors and are validated for clinical decision-making, the incremental predictive value of hepatic steatosis indices beyond such models may be limited. Therefore, their practical utility may lie in identifying individuals at low risk (risk de-escalation) rather than definitive risk classification.

These indices were originally developed for the purpose of screening for non-alcoholic fatty liver disease (NAFLD), and prior studies have shown that each is a useful tool reflecting the presence of NAFLD to some extent. Reported AUCs for NAFLD diagnosis include 0.822 for the ZJU index, [12] 0.84–0.870 for FLI, [14] 0.812 for HSI, [13] and 0.929 for K-NAFLD score, [15] indicating their utility as effective noninvasive screening tools. Although these indices were developed under the NAFLD framework, they are currently often applied as surrogate markers of hepatic steatosis in populations that largely correspond to MASLD.

In comparative analyses, the K-NAFLD score demonstrated significantly higher AUCs for predicting NAFLD than FLI (difference: –0.051) and HSI (–0.282), with all *p*-values < 0.001, further supporting its superior predictive ability for NAFLD [15]

A close association between MASLD and CVD has been well established [24] The FLI has been correlated with CVD incidence in the general population [17] Although no prior studies have demonstrated a significant association between the HSI and cardiovascular events in the general population, such association has been reported in patients with hypertension and obstructive sleep apnea [25]

In addition, patients diagnosed with hepatic steatosis using the K-NAFLD score have shown increased CVD risk, particularly in the presence of metabolic dysfunction [16] Although the ZJU index has been associated with hypertension, no direct association with cardiovascular events has been reported to date [26]

In this study, differences in the predictive ability for cardiovascular events were observed among the hepatic steatosis indices, with the FLI and K-NAFLD score demonstrating a relatively higher predictive performance than the ZJU index and HSI. These differences may be partly attributable to variations in the components of each index (Supplemental Table 1). Among these components, the presence or absence of WC appears to play a key role in predictive accuracy.

WC reflects visceral fat accumulation and ectopic fat deposition, both of which are associated with increased CVD risk [[27], [28], [29]] Notably, a high prevalence of “lean MASLD (formerly referred to as “lean NAFLD”)” has been reported among Asian populations, in which individuals may have a normal BMI but still exhibit visceral adiposity and metabolic abnormalities [[30], [31], [32], [33]]Both the FLI and K-NAFLD score include WC as a component, suggesting that their ability to capture these Asian-specific fat distribution patterns may have contributed to their superior predictive ability for cardiovascular events.

The K-NAFLD score was originally developed based on a Korean cohort study; however, it demonstrated good predictive performance in the Japanese population as well. This may reflect shared characteristics among East Asian populations, such as body composition, insulin resistance, and dietary habits.

However, the extent to which these findings can be generalized to non-Asian populations remains uncertain. External validation and direct comparison in Western cohorts will be important to determine whether differences in body composition and fat distribution modify the predictive performance and optimal cutoffs of these indices.

Beyond their original purpose of detecting hepatic steatosis, hepatic steatosis indices may also serve as practical tools for cardiovascular risk stratification [34] These indices incorporate metabolic parameters such as triglycerides, liver enzymes, and anthropometric measures that are routinely available in health checkups. Accordingly, they may capture subclinical metabolic dysfunction and systemic inflammation that are not fully reflected by traditional cardiovascular risk factors. Given the increasing burden of both steatotic liver disease and CVD, particularly in aging populations, the use of hepatic steatosis indices could provide a simple and cost-effective approach for dual-purpose risk assessment in clinical and public health settings.

The ZJU index showed higher sensitivity under high-specificity conditions, implying its suitability in contexts where false positives must be minimized. In contrast, the K-NAFLD score and FLI had higher sensitivity at their respective optimal cutoff points, supporting their utility in broader screening strategies. These findings emphasize the importance of selecting an appropriate index based on the clinical or public health setting.

In the subgroup analysis of participants aged ≥50 years, the results were generally consistent with those of the overall analysis. However, the HSI was not significantly associated with the incidence of CVD in this subgroup. In older adults, the presence of metabolic abnormalities may exert a stronger influence on the development of CAD and stroke. The HSI does not include direct markers of metabolic dysfunction in its components, which may explain its limited predictive ability in this age group.

In the additional analysis focusing on participants with a higher cardiovascular risk profile, the AUC values of the hepatic steatosis indices tended to be lower than those observed in the overall population. This may reflect the fact that individuals in the high-risk group are influenced by multiple cardiometabolic factors beyond hepatic steatosis, thereby reducing the relative contribution of hepatic steatosis indices to overall cardiovascular risk prediction.

The Fibrosis-4 (Fib-4) index was originally developed to predict liver fibrosis in patients with HIV/HCV coinfection and is calculated based on four variables: AST, ALT, platelet count, and age [35] Although it was initially intended for use in this specific population, subsequent studies have shown that FIB-4 is useful for stratifying the risk of advanced liver fibrosis and severe liver-related outcomes across broader clinical settings [36]

Moreover, the Fib-4 index has been associated with the incidence of cardiovascular events and CVD-related mortality in populations with obesity or diabetes, [37,38] suggesting its potential applicability beyond liver-specific risk assessment. However, as platelet counts are not routinely measured during general health checkups in Japan, the FIB-4 index was not included in the present analysis. Future studies directly comparing hepatic steatosis indices with fibrosis-based scores such as FIB-4 may provide further insight into their relative value for cardiovascular risk stratification.

One limitation of this study is that it was conducted within a single occupational cohort of Japanese individuals. Therefore, further studies are warranted to validate these findings and to examine potential ethnic/regional differences in predictive performance and optimal cutoffs, including comparisons with Western populations. Moreover, because this was an occupational cohort, most participants were middle-aged and relatively few older adults were included. Therefore, the predictive performance of these indices in older populations could not be adequately evaluated. In addition, hepatic steatosis was assessed using surrogate indices and was not confirmed by imaging modalities such as ultrasonography; therefore, some degree of misclassification is possible, and we could not evaluate index performance according to imaging-defined steatosis status. Furthermore, participants could not be classified according to the presence or absence of imaging-defined hepatic steatosis. Another limitation is that CAD and stroke events were identified through self-administered questionnaires, which may be subject to recall bias or misclassification, including potential underreporting of non-fatal events. For instance, participants may have failed to report events that occurred during follow-up, misunderstood medical diagnoses, or inaccurately reported the timing or type of cardiovascular events. In particular, angina pectoris represents a relatively soft coronary endpoint and may be subject to diagnostic variability. Such inaccuracies could result in the underestimation or misclassification of outcomes. In addition, a substantial number of participants were excluded due to missing data or loss to follow-up, which was primarily attributable to retirement from the occupational cohort. This may have introduced selection bias. Compared with included participants, excluded individuals tended to be older, less likely to be male, and had a higher prevalence of medication use for diabetes, hypertension, and dyslipidemia (Supplemental Table 14). Although many of these differences reached statistical significance owing to the large sample size, the absolute between-group differences were small in magnitude. Nevertheless, this potential selection bias should be considered when interpreting the results.

## 5 Conclusion

In this study, we compared the predictive abilities of four hepatic steatosis indices (the ZJU index, HSI, FLI, and K-NAFLD score) for cardiovascular events in a general Japanese population. Our findings demonstrated that the FLI and K-NAFLD score had superior predictive performance to the other indices. These results suggest that noninvasive indices for steatotic liver disease, particularly the FLI and K-NAFLD score, may also be useful for early risk assessment of CVD, providing a foundation for their potential application in both public health and clinical settings.

## Funding sources

This research was supported by MHLW Comprehensive Research Project for Measures against Cardiovascular Diseases, Diabetes and Other Lifestyle Related Diseases Program, Grant Number JPMH 24FA1008. The sponsor was not involved in the collection, analysis and interpretation of data, and in the writing of the article.

## Declaration of generative AI and AI-assisted technologies in the writing process

During the preparation of this work the author used ChatGPT in order to improve the English language and readability of the manuscript. After using this tool/service, the author reviewed and edited the content as needed and takes full responsibility for the content of the publication.

**Supplemental Figure 1. Calibration plots for prediction of 10-year cardiovascular events.** Participants were divided into 20 groups according to predicted risk from each model. The mean predicted risk within each group was plotted against the observed 10-year event probability estimated using Kaplan–Meier methods. The dashed diagonal line represents perfect calibration. The base model included age, sex, body mass index, smoking status, physical activity, alcohol intake, HDL cholesterol, LDL cholesterol, and medication use for hypertension, dyslipidemia, and diabetes. Each hepatic steatosis index (ZJU index, hepatic steatosis index [HSI], fatty liver index [FLI], and K-NAFLD score) was added individually to the base model.

## CRediT authorship contribution statement

**Nozomi Kuramoto:** Writing – original draft, Visualization, Methodology, Investigation, Formal analysis. **Hiroshi Okada:** Writing – review & editing, Validation, Software, Project administration, Methodology, Investigation, Formal analysis, Data curation, Conceptualization. **Hanako Nakajima:** Writing – review & editing, Investigation. **Naoko Nakanishi:** Writing – review & editing, Investigation. **Emi Ushigome:** Writing – review & editing, Investigation. **Masahide Hamaguchi:** Writing – review & editing, Investigation. **Toshiaki Ohkuma:** Writing – review & editing, Resources, Investigation. **Daisuke Tanaka:** Writing – review & editing, Resources, Investigation. **Teruki Miyake:** Writing – review & editing, Resources, Investigation. **Kazushiro Kurogi:** Writing – review & editing, Resources, Investigation. **Hiroaki Murata:** Writing – review & editing, Resources, Investigation. **Eri Tsuda:** Writing – review & editing, Resources, Investigation. **Naoki Yoshida:** Writing – review & editing, Resources, Investigation. **Masato Ito:** Writing – review & editing, Resources, Investigation. **Michiaki Fukui:** Writing – review & editing, Supervision, Investigation.

## Declaration of competing interest

The authors declare the following financial interests/personal relationships which may be considered as potential competing interests:

Hiroshi Okada reports financial support was provided by Japan Diabetes Foundation. Emi Ushigome reports financial support was provided by Yoshinoya Holdings Co., Ltd. Emi Ushigome reports financial support was provided by Taiyo Kagaku Co., Ltd. Masahide Hamaguchi reports financial support was provided by AstraZeneca K.K. Masahide Hamaguchi reports financial support was provided by Ono Pharma Co. Ltd. Masahide Hamaguchi reports financial support was provided by Kowa Pharma Co. Ltd. Michiaki Fukui reports financial support was provided by Ono Pharma Co. Ltd. Michiaki Fukui reports financial support was provided by Oishi Kenko inc. Michiaki Fukui reports financial support was provided by Yamada Bee Farm. Michiaki Fukui reports financial support was provided by Nippon Boehringer Ingelheim Co. Ltd. Michiaki Fukui reports financial support was provided by Kissei Pharma Co. Ltd. Michiaki Fukui reports financial support was provided by Mitsubishi Tanabe Pharma Corp. Michiaki Fukui reports financial support was provided by Daiichi Sankyo Co. Ltd. Michiaki Fukui reports financial support was provided by Sanofi K.K. Michiaki Fukui reports financial support was provided by Takeda Pharma Co. Ltd. Michiaki Fukui reports financial support was provided by Astellas Pharma Inc. Michiaki Fukui reports financial support was provided by MSD K.K. Michiaki Fukui reports financial support was provided by Kyowa Kirin Co., Ltd. Michiaki Fukui reports financial support was provided by Sumitomo Dainippon Pharma Co., Ltd. Michiaki Fukui reports financial support was provided by Kowa Pharma Co. Ltd. Michiaki Fukui reports financial support was provided by Novo Nordisk Pharma Ltd. Michiaki Fukui reports financial support was provided by Sanwa Kagaku Kenkyusho CO., Ltd. Michiaki Fukui reports financial support was provided by Eli Lilly, Japan, K.K. Michiaki Fukui reports financial support was provided by Taisho Pharma Co., Ltd. Michiaki Fukui reports financial support was provided by Terumo Corp. Michiaki Fukui reports financial support was provided by Tejin Pharma Ltd. Michiaki Fukui reports financial support was provided by Nippon Chemiphar Co. Ltd. Michiaki Fukui reports financial support was provided by Abbott Japan Co. Ltd. Michiaki Fukui reports financial support was provided by Johnson & Johnson K.K. Medical Co. If there are other authors, they declare that they have no known competing financial interests or personal relationships that could have appeared to influence the work reported in this paper.

## Appendix Supplementary materials

Image, application 1

Image, application 2
