# Supplementary material for: Steatotic liver disease indices for cardiovascular event prediction: Panasonic cohort study 28
Source: Am J Prev Cardiol. 2026 Mar 10;27:101541. doi: 10.1016/j.ajpc.2026.101541 (PMC13261237; doi:10.1016/j.ajpc.2026.101541)
Supplement: Supplementary file 1 [file mmc1.docx]

Supplemental Table 1. Components included in each steatotic liver disease index

| Variable | ZJU index | HSI | FLI | K-NAFLD score |
| --- | --- | --- | --- | --- |
| Body mass index (BMI) | ✓ | ✓ | ✓ | - |
| Waist circumference (WC) | - | - | ✓ | ✓ |
| Triglycerides (TG) | ✓ | - | ✓ | ✓ |
| Gamma-glutamyl transferase (GGT) | - | - | ✓ | - |
| Alanine aminotransferase (ALT) | - | - | - | ✓ |
| Aspartate aminotransferase (AST) | - | - | - | - |
| ALT/AST ratio | ✓ | ✓ | - | - |
| Fasting plasma glucose (FPG) | ✓ | - | - | ✓ |
| Systolic blood pressure (SBP) | - | - | - | ✓ |
| Sex term | ✓ | ✓ | - | ✓ |
| Diabetes mellitus term | - | ✓ | - | - |

✓ indicates that the variable is incorporated into the corresponding index formula.

Supplemental Table 2. Multivariable-adjusted hazard ratios additionally adjusted for sex.

| MACE | aHR | 95% CI |
| --- | --- | --- |
| ZJU index (per SD) | 1.14 | (1.10–1.18) |
| logHSI (per SD) | 1.08 | (1.04–1.12) |
| logFLI (per SD) | 1.19 | (1.13–1.25) |
| logK-NAFLD score (per SD) | 1.18 | (1.13–1.22) |
| CAD | aHR | 95% CI |
| ZJU index (per SD) | 1.14 | (1.09–1.19) |
| logHSI (per SD) | 1.09 | (1.04–1.14) |
| logFLI (per SD) | 1.21 | (1.14–1.29) |
| logK-NAFLD score (per SD) | 1.20 | (1.14–1.25) |
| Stroke | aHR | 95% CI |
| ZJU index (per SD) | 1.13 | (1.06–1.20) |
| logHSI (per SD) | 1.07 | (1.00–1.14) |
| logFLI (per SD) | 1.15 | (1.05–1.25) |
| logK-NAFLD score (per SD) | 1.14 | (1.06–1.22) |

The multivariate model was adjusted for age, smoking status, alcohol consumption, physical activity, HDL cholesterol, LDL cholesterol, use of diabetes medication, use of antihypertensive medication, and use of dyslipidemia medication.

Abbreviations: MACE, major adverse cardiovascular event; CAD, coronary artery disease; aHR, adjusted hazard ratio; CI, confidence interval; ZJU index, Zhejiang University Index; HSI, Hepatic Steatosis Index; FLI, Fatty Liver Index; K-NAFLD score, Korean National Health and Nutrition Examination Survey Non-Alcoholic Fatty Liver Disease Score.

Supplemental Table 3. Likelihood ratio tests for model improvement

| **MACE** | **-2LL_0** | **-2LL_1** | **AIU** | **-2(LL_0-LL_1)** | **df** | **p-value** |
| --- | --- | --- | --- | --- | --- | --- |
| ZJU index | 78429.8 | 78389.9 | 78412.0 | 39.84 | 1 | <0.001 |
| HSI |  | 78414.4 | 78436.4 | 15.36 | 1 | <0.001 |
| FLI |  | 78356.6 | 78378.6 | 73.19 | 1 | <0.001 |
| K-NAFLD score |  | 78402.1 | 78424.1 | 27.68 | 1 | <0.001 |
| **CAD** | **-2LL_0** | **-2LL_1** | **AIU** | **-2(LL_0-LL_1)** | **df** | **p-value** |
| ZJU index | 53292.2 | 53263.0 | 53285.0 | 29.21 | 1 | <0.001 |
| HSI |  | 53279.8 | 53301.8 | 12.39 | 1 | <0.001 |
| FLI |  | 53231.3 | 53253.3 | 60.87 | 1 | <0.001 |
| K-NAFLD score |  | 53268.7 | 53290.7 | 23.53 | 1 | <0.001 |
| **Stroke** | **-2LL_0** | **-2LL_1** | **AIU** | **-2(LL_0-LL_1)** | **df** | **p-value** |
| ZJU index | 26164.0 | 26152.2 | 26174.2 | 11.80 | 1 | <0.001 |
| HSI |  | 26159.8 | 26181.8 | 4.26 | 1 | 0.039 |
| FLI |  | 26152.5 | 26174.5 | 11.54 | 1 | <0.001 |
| K-NAFLD score |  | 26158.3 | 26180.3 | 5.74 | 1 | 0.017 |

Likelihood ratio tests were conducted to evaluate whether adding each hepatic steatosis index to the base model significantly improved prediction of cardiovascular outcomes. The base model included age, HDL cholesterol, LDL cholesterol, use of antihypertensive medication, use of dyslipidemia medication, use of diabetes medication, alcohol consumption, physical activity, and smoking status.
**-2LL₀:** The -2 log-likelihood of the model with the seven covariates
**-2LL₁:** The -2 log-likelihood of the model with the above seven covariates plus each fatty liver index
**-2(LL₀−LL₁):** Test statistic
**df:** Degrees of freedom of the test statistic (difference in the number of parameters)

**Abbreviations:** MACE, major adverse cardiovascular event; CAD, coronary artery disease; ZJU index, Zhejiang University Index; HSI, Hepatic Steatosis Index; FLI, Fatty Liver Index; K-NAFLD score, Korean National Health and Nutrition Examination Survey Non-Alcoholic Fatty Liver Disease Score.

Supplemental Table 4. Cut-off values for predicting major adverse cardiovascular events

|  | Model | Cut-off value  (95% CI) | Sensitivity  (95% CI) | Specificity  (95% CI) | NPV  (95% CI) | PPV  (95% CI) | NLR  (95% CI) | PLR  (95% CI) |
| --- | --- | --- | --- | --- | --- | --- | --- | --- |
| cutoff value | ZJU-index | 32.94  (32.00–34.20) | 61.8%  (50.8–70.0) | 52.4%  (44.1–63.0) | 96.8%  (96.5–97.0) | 5.6%  (5.4–6.1) | 0.73  (0.68–0.79) | 1.30  (1.25–1.40) |
|  | HSI | 32.12  (30.90–32.80) | 55.8%  (50.3–64.5) | 56.1%  (47.4–61.0) | 96.5%  (96.3–96.7) | 5.5%  (5.2–5.8) | 0.79  (0.75–0.82) | 1.27  (1.22–1.32) |
|  | FLI | 23.33  (16.00–32.00) | 61.2%  (50.9–71.8) | 55.8%  (45.7–66.0) | 96.9%  (96.7–97.3) | 6.0%  (5.5–6.5) | 0.70  (0.62–0.75) | 1.39  (1.30–1.52) |
|  | K-NAFLD score | -2.61  (-3.10–-1.80) | 63.3%  (49.9–71.0) | 54.1%  (46.5–66.7) | 97.0%  (96.6–97.3) | 5.9%  (5.6–6.6) | 0.68  (0.62–0.75) | 1.38  (1.31–1.53) |
| 70% specificity | ZJU-index | 35.24  (35.18–35.34) | 42.4%  (40.3–43.9) | 70.0%  (70.0–70.7) | 96.4%  (96.2–96.5) | 6.1%  (5.8–6.4) | 0.82  (0.80–0.85) | 1.41  (1.36–1.48) |
|  | HSI | 34.38  (34.30–34.41) | 40.1%  (38.2–41.5) | 70.0%  (70.0–70.5) | 96.2%  (96.1–96.4) | 5.8%  (5.5–6.1) | 0.86  (0.83–0.88) | 1.34  (1.28–1.40) |
|  | FLI | 37.04  (36.96–37.08) | 45.4%  (43.3–46.6) | 70.0%  (70.0–70.7) | 96.6%  (96.4–96.7) | 6.5%  (6.2–6.8) | 0.78  (0.76–0.81) | 1.51  (1.46–1.58) |
|  | K-NAFLD score | -1.45  (-1.49–-1.41) | 45.5%  (42.8–47.1) | 70.1%  (70.0–71.1) | 96.6%  (96.4–96.7) | 6.5%  (6.2–6.8) | 0.78  (0.75–0.81) | 1.52  (1.46–1.58) |
| 80% specificity | ZJU-index | 37.00  36.99–37.10) | 31.2%  (29.3–32.5) | 80.0%  (80.0–80.4) | 96.2%  (96.0–96.4) | 6.7%  (6.3–7.1) | 0.86  (0.84–0.88) | 1.56  (1.48–1.64) |
|  | HSI | 36.50  (36.45–36.56) | 28.6%  (26.9–29.9) | 80.0%  (80.0–80.4) | 96.1%  (95.9–96.2) | 6.1%  (5.8–6.5) | 0.89  (0.87–0.91) | 1.43  (1.36–1.51) |
|  | FLI | 50.57  (50.02–51.03) | 32.8%  (30.4–34.1) | 80.0%  (80.0–80.6) | 96.3%  (96.1–96.4) | 7.0%  (6.6–7.4) | 0.84  (0.82–0.87) | 1.64  (1.56–1.73) |
|  | K-NAFLD score | -0.39  (-0.43–-0.36) | 32.6%  (30.5–33.6) | 80.0%  (80.0–80.5) | 96.3%  (96.1–96.4) | 6.9%  (6.5–7.3) | 0.84  (0.83–0.87) | 1.63  (1.54–1.71) |
| 90% specificity | ZJU-index | 39.77  (39.70–39.81) | 18.0%  (16.3–19.2) | 90.0%  (90.0–90.3) | 96.0%  (95.8–96.1) | 7.6%  (7.0–8.3) | 0.91  (0.90–0.93) | 1.81  (1.66–1.95) |
|  | HSI | 39.87  (39.82–39.92) | 15.2%  (13.9–16.3) | 90.0%  (90.0–90.2) | 95.9%  (95.7–96.0) | 6.5%  (6.0–7.1) | 0.94  (0.93–0.96) | 1.52  (1.41–1.65) |
|  | FLI | 68.95  (68.98–69.02) | 18.1%  (16.5–19.1) | 90.0%  (90.0–90.4) | 96.0%  (95.8–96.2) | 7.7%  (7.1–8.3) | 0.91  (0.90–0.93) | 1.82  (1.68–1.96) |
|  | K-NAFLD score | 1.51  (1.50–1.56) | 16.7%  (15.2–17.9) | 90.0%  (90.0–90.4) | 95.9%  (95.8–96.1) | 7.1%  (6.6–7.7) | 0.93  (0.91–0.94) | 1.68  (1.54–1.82) |

Cut-off values for each hepatic steatosis index were determined using time-dependent ROC analysis for 10-year prediction of major adverse cardiovascular events. Additional thresholds were set by fixing specificity at 70%, 80%, and 90%, with corresponding sensitivity, specificity, negative predictive value (NPV), positive predictive value (PPV), negative likelihood ratio (NLR), and positive likelihood ratio (PLR) calculated.

Abbreviations: MACE, major adverse cardiovascular events; ZJU index, Zhejiang University Index; HSI, Hepatic Steatosis Index; FLI, Fatty Liver Index; K-NAFLD score, Korean National Health and Nutrition Examination Survey Non-Alcoholic Fatty Liver Disease Score.

Supplemental Table 5. Cut-off values for predicting coronary artery disease

|  | Model | Cut-off value | Sensitivity | Specificity | NPV | PPV | NLR | PLR |
| --- | --- | --- | --- | --- | --- | --- | --- | --- |
| cutoff value | ZJU-index | 33.97  (32.01–34.53) | 54.3%  (49.2–70.4) | 60.8%  (44.0–65.1) | 97.7%  (97.6–98.0) | 4.2%  (3.8–4.5) | 0.75  (0.66–0.78) | 1.38  (1.26–1.47) |
|  | HSI | 32.12  (31.46–34.48) | 56.9%  (42.1–60.7) | 56.0%  (51.9–70.8) | 97.6%  (97.4–97.8) | 3.9%  (3.7–4.4) | 0.77  (0.73–0.83) | 1.29  (1.24–1.44) |
|  | FLI | 23.33  (17.01–31.98) | 62.7%  (52.1–70.3) | 55.6%  (47.3–65.8) | 97.9%  (97.7–98.1) | 4.3%  (4.0–4.7) | 0.67  (0.62–0.73) | 1.41  (1.34–1.54) |
|  | K-NAFLD score | -2.61  (-3.01–-1.72) | 64.0%  (49.0–70.8) | 53.9%  (48.0–68.0) | 97.9%  (97.6–98.1) | 4.2%  (4.0–4.9) | 0.67  (0.61–0.75) | 1.39  (1.32–1.61) |
| 70% specificity | ZJU-index | 35.26  (35.16–35.33) | 43.5%  (41.0–45.3) | 70.0%  (70.0–70.7) | 97.5%  (97.4–97.6) | 4.4%  (4.1–4.7) | 0.81  (0.78–0.84) | 1.45  (1.39–1.53) |
|  | HSI | 34.40  (34.30–34.44) | 41.7%  (39.5–43.3) | 70.1%  (70.0–70.5) | 97.4%  (97.3–97.6) | 4.2%  (4.0–4.5) | 0.83  (0.81–0.86) | 1.39  (1.33–1.46) |
|  | FLI | 37.23  (36.95–38.01) | 46.4%  (43.9–48.0) | 70.0%  (70.0–70.8) | 97.6%  (97.3–97.8) | 4.7%  (4.4–4.9) | 0.77  (0.74–0.80) | 1.55  (1.48–1.62) |
|  | K-NAFLD score | -1.44  (-1.53–-1.36) | 46.8%  (43.7–48.5) | 70.0%  (70.0–71.2) | 97.7%  (97.5–97.8) | 4.7%  (4.5–5.0) | 0.76  (0.73–0.79) | 1.56  (1.51–1.64) |
| 80% specificity | ZJU-index | 37.02  (36.98–37.11) | 32.0%  (29.6–33.6) | 80.0%  (80.0–80.5) | 97.4%  (97.2–97.5) | 4.8%  (4.5–5.1) | 0.85  (0.83–0.88) | 1.60  (1.50–1.70) |
|  | HSI | 36.53  (36.45–36.60) | 29.3%  (27.2–31.1) | 80.0%  (80.0–80.4) | 97.3%  (97.1–97.4) | 4.4%  (4.1–4.8) | 0.88  (0.86–0.91) | 1.47  (1.38–1.57) |
|  | FLI | 50.80  (49.98–51.02) | 33.5%  (30.9–34.8) | 80.0%  (80.0–80.6) | 97.4%  (97.3–97.6) | 5.0%  (4.7–5.4) | 0.83  (0.81–0.86) | 1.67  (1.58–1.77) |
|  | K-NAFLD score | -0.37  (-0.44–-0.34) | 33.9%  (31.2–35.5) | 80.0%  (80.0–80.7) | 97.5%  (97.3–97.6) | 5.1%  (4.7–5.4) | 0.83  (0.80–0.85) | 1.70  (1.59–1.80) |
| 90% specificity | ZJU-index | 39.80  (39.72–39.90) | 18.2%  (16.4–19.8) | 90.0%  (90.0–90.3) | 97.2%  (97.1–97.3) | 5.4%  (5.0–6.0) | 0.91  (0.89–0.93) | 1.82  (1.66–2.00) |
|  | HSI | 39.90  (39.77–40.01) | 15.5%  (13.9–16.9) | 90.0%  (90.0–90.2) | 97.1%  (97.0–97.2) | 4.7%  (4.2–5.1) | 0.94  (0.92–0.96) | 1.56  (1.40–1.71) |
|  | FLI | 69.17  (69.00–69.99) | 18.4%  (16.5–19.6) | 90.0%  (90.0–90.4) | 97.2%  (97.1–97.3) | 5.5%  (5.0–6.0) | 0.91  (0.89–0.93) | 1.84  (1.67–1.99) |
|  | K-NAFLD score | 1.53  (1.50–1.60) | 17.0%  (15.1–18.5) | 90.0%  (90.0–90.4) | 97.2%  (97.0–97.3) | 5.1%  (4.6–5.6) | 0.92  (0.90–0.94) | 1.70  (1.55–1.87) |

Cut-off values for each hepatic steatosis index were determined using time-dependent ROC analysis for 10-year prediction of coronary artery disease. Additional thresholds were set by fixing specificity at 70%, 80%, and 90%, with corresponding sensitivity, specificity, negative predictive value (NPV), positive predictive value (PPV), negative likelihood ratio (NLR), and positive likelihood ratio (PLR) calculated.

Abbreviations: CAD, coronary artery disease; ZJU index, Zhejiang University Index; HSI, Hepatic Steatosis Index; FLI, Fatty Liver Index; K-NAFLD score, Korean National Health and Nutrition Examination Survey Non-Alcoholic Fatty Liver Disease Score.

Supplemental Table 6. Cut-off values for predicting stroke

|  | Model | Cut-off value | Sensitivity | Specificity | NPV | PPV | NLR | PLR |
| --- | --- | --- | --- | --- | --- | --- | --- | --- |
| cutoff value | ZJU-index | 32.94  (31.89–35.41) | 60.9%  (42.1–70.3) | 51.9%  (42.7–70.8) | 98.9%  (98.8–99.0) | 1.8%  (1.6–2.0) | 0.75  (0.69–0.83) | 1.27  (1.20–1.42) |
|  | HSI | 32.38  (29.01–33.63) | 52.4%  (44.3–77.8) | 57.5%  (31.7–65.4) | 98.8%  (98.7–99.1) | 1.7%  (1.5–1.9) | 0.83  (0.66–0.86) | 1.23  (1.13–1.32) |
|  | FLI | 16.78  (10.98–32.02) | 69.6%  (49.1–78.3) | 45.9%  (36.1–65.5) | 99.1%  (98.9–99.2) | 1.8%  (1.7–2.1) | 0.66  (0.58–0.78) | 1.29  (1.23–1.49) |
|  | K-NAFLD score | -2.67  (-3.51–-2.49) | 63.1%  (58.5–77.2) | 52.6%  (38.8–56.4) | 99.0%  (98.9–99.2) | 1.9%  (1.7–2.0) | 0.70  (0.58–0.75) | 1.33  (1.23–1.41) |
| 70% specificity | ZJU-index | 35.30  (35.28–35.34) | 40.8%  (37.5–43.6) | 70.0%  (70.0–70.5) | 98.8%  (98.7–98.9) | 1.9%  (1.7–2.1) | 0.85  (0.80–0.89) | 1.36  (1.26–1.47) |
|  | HSI | 34.44  (34.37–34.52) | 36.5%  (33.5–39.2) | 70.0%  (70.0–70.6) | 98.7%  (98.6–98.8) | 1.7%  (1.5–1.9) | 0.91  (0.87–0.95) | 1.22  (1.13–1.32) |
|  | FLI | 37.62  (37.03–38.04) | 41.6%  (38.2–44.7) | 70.0%  (70.0–70.8) | 98.8%  (98.7–98.9) | 1.9%  (1.8–2.1) | 0.83  (0.79–0.88) | 1.39  (1.30–1.51) |
|  | K-NAFLD score | -1.41  (-1.42–-1.40) | 42.6%  (38.5–44.5) | 70.1%  (70.0–71.0) | 98.8%  (98.7–98.9) | 2.0%  (1.8–2.2) | 0.82  (0.78–0.87) | 1.42  (1.32–1.53) |
| 80% specificity | ZJU-index | 37.08  (37.01–37.10) | 28.5%  (25.0–30.9) | 80.0%  (80.0–80.4) | 98.7%  (98.6–98.8) | 2.0%  (1.7–2.2) | 0.89  (0.86–0.93) | 1.43  (1.27–1.56) |
|  | HSI | 36.58  (36.48–36.64) | 27.1%  (24.2–29.7) | 80.0%  (80.0–80.4) | 98.7%  (98.6–98.8) | 1.9%  (1.7–2.1) | 0.91  (0.88–0.95) | 1.36  (1.22–1.50) |
|  | FLI | 51.24  (51.02–52.03) | 29.6%  (26.5–32.3) | 80.0%  (80.0–80.6) | 98.8%  (98.7–98.9) | 2.1%  (1.8–2.3) | 0.88  (0.84–0.92) | 1.48  (1.33–1.63) |
|  | K-NAFLD score | -0.33  (-0.44–-0.34) | 29.4%  (25.9–31.6) | 80.0%  (80.0–80.7) | 98.8%  (98.7–99.0) | 2.1%  (1.8–2.3) | 0.88  (0.85–0.92) | 1.48  (1.33–1.62) |
| 90% specificity | ZJU-index | 39.86  (39.77–39.93) | 17.0%  (14.6–19.1) | 90.0%  (90.0–90.3) | 98.7%  (98.6–98.8) | 2.4%  (2.0–2.7) | 0.92  (0.90–0.95) | 1.70  (1.49–1.95) |
|  | HSI | 39.94  (39.90–40.14) | 13.9%  (11.8–16.0) | 90.0%  (90.0–90.4) | 98.7%  (98.6–98.9) | 1.9%  (1.6–2.3) | 0.96  (0.93–0.98) | 1.39  (1.19–1.63) |
|  | FLI | 69.53  (69.05–70.00) | 16.8%  (14.2–18.7) | 90.0%  (90.0–90.5) | 98.7%  (98.6–98.8) | 2.3%  (2.0–2.7) | 0.93  (0.90–0.95) | 1.68  (1.47–1.91) |
|  | K-NAFLD score | 1.57  (1.51–1.66) | 15.7%  (13.2–17.6) | 90.0%  (90.0–90.4) | 98.7%  (98.6–98.8) | 2.2%  (1.9–2.5) | 0.94  (0.91–0.96) | 1.57  (1.35–1.79) |

Cut-off values for each hepatic steatosis index were determined using time-dependent ROC analysis for 10-year prediction of stroke. Additional thresholds were set by fixing specificity at 70%, 80%, and 90%, with corresponding sensitivity, specificity, negative predictive value (NPV), positive predictive value (PPV), negative likelihood ratio (NLR), and positive likelihood ratio (PLR) calculated.

Abbreviations: ZJU index, Zhejiang University Index; HSI, Hepatic Steatosis Index; FLI, Fatty Liver Index; K-NAFLD score, Korean National Health and Nutrition Examination Survey Non-Alcoholic Fatty Liver Disease Score.Supplemental Table 7. Integrated discrimination improvement (IDI) and continuous net reclassification improvement (cNRI) for each hepatic steatosis index compared with the base model for prediction of MACE.

| Comparison | IDI (95% CI) | cNRI (95% CI) |
| --- | --- | --- |
| Base vs ZJU-index | 0.00010 (−0.00002 to 0.00041) | 0.005 (−0.029 to 0.041) |
| Base vs HSI | 0.00017 (0.00010 to 0.00040) | 0.016 (−0.017 to 0.052) |
| Base vs FLI | 0.00003 (−0.00008 to 0.00034) | 0.074 (0.036 to 0.113) |
| Base vs K-NAFLD | 0.00038 (0.00008 to 0.00086) | 0.083 (0.046 to 0.121) |

The base model included age, sex, body mass index, smoking status, physical activity, alcohol intake, HDL cholesterol, LDL cholesterol, and medication use for hypertension, dyslipidemia, and diabetes. Values are shown as point estimates with 95% confidence intervals.

Abbreviations: ZJU index, Zhejiang University Index; HSI, Hepatic Steatosis Index; FLI, Fatty Liver Index; K-NAFLD score, Korean National Health and Nutrition Examination Survey Non-Alcoholic Fatty Liver Disease Score; IDI, integrated discrimination improvement; cNRI, continuous net reclassification improvement; MACE, major adverse cardiovascular events.

Supplemental Table 8. Baseline characteristics of participants aged ≥50 years (subgroup analysis)

| N | 82225 |
| --- | --- |
| Age(y) | 50(50-53) |
| BMI(kg/m²) | 23.2(21.3-25.4) |
| Height(cm) | 169.1(163.8-173.6) |
| WC(cm) | 83.2(77.7-89.2) |
| ZJU-index | 33.2(30.6-36.5) |
| HIS | 31.7(28.6-35.5) |
| FLI | 24.3(10.1-48.5) |
| K-NAFLD score | -2.4(-3.7--0.6) |
| Systolic blood pressure(mmHg) | 122.0(112.0-130.0) |
| Diastolic blood pressure(mmHg) | 78.0(70.0-84.0) |
| Fasting plasma glucose(mg/dL) | 94.0(89.0-102.0) |
| Tryglycerides(mg/dL) | 97.0(68.0-143.0) |
| HDL cholesterol(mg/dL) | 58.0(49.0-70.0) |
| LDL cholesterol(mg/dL) | 127.0(107.0-147.0) |
| AST(IU/L) | 21.0(18.0-26.0) |
| ALT(IU/L) | 21.0(15.0-30.0) |
| GGT(IU/L) | 31.0(21.0-53.0) |
| smoking(none/past/current),n,(%) | 41172/16511/24540 (50.0/20.0/30.0) |
| Alcohol consumption,n,(+)(%) | 20482 (25.0) |
| Physical exercise,n,(+)(%) | 17596 (21.4) |
| Diabetes medication use, n, (+)(%) | 3376 (4.1) |
| Antihypertensive medication use, n, (+)(%) | 11847 (14.4) |
| Dyslipidemia medication use, n, (+)(%) | 5056 (6.1) |

Data are presented as mean (standard deviation) or absolute number (percentage).

Abbreviations: BMI, body mass index; WC, waist circumference; ZJU index, Zhejiang University Index; HSI, Hepatic Steatosis Index; FLI, Fatty Liver Index; K-NAFLD score, Korean National Health and Nutrition Examination Survey Non-Alcoholic Fatty Liver Disease Score; LDL, low-density lipoprotein; HDL, high-density lipoprotein.

Supplemental Table 9. Multivariable-adjusted hazard ratios for participants aged ≥50 years

| **MACE** | aHR | 95% CI |
| --- | --- | --- |
| ZJU index (per SD) | 1.07 | (1.02, 1.12) |
| logHSI (per SD) | 1.03 | (0.98, 1.08) |
| logFLI (per SD) | 1.15 | (1.09, 1.22) |
| logK-NAFLD score (per SD) | 1.14 | (1.09, 1.20) |
| **CAD** | aHR | 95% CI |
| ZJU index (per SD) | 1.06 | (1.00, 1.12) |
| logHSI (per SD) | 1.02 | (0.97, 1.08) |
| logFLI (per SD) | 1.16 | (1.09, 1.24) |
| logK-NAFLD score (per SD) | 1.14 | (1.08, 1.20) |
| **Stroke** | aHR | 95% CI |
| ZJU index (per SD) | 1.08 | (1.00, 1.17) |
| logHSI (per SD) | 1.03 | (0.95, 1.12) |
| logFLI (per SD) | 1.15 | (1.05, 1.27) |
| logK-NAFLD score (per SD) | 1.14 | (1.05, 1.24) |

The multivariate model was adjusted for age, smoking status, alcohol consumption, physical activity, HDL cholesterol, LDL cholesterol, use of diabetes medication, use of antihypertensive medication, and use of dyslipidemia medication.

Abbreviations: MACE, major adverse cardiovascular event; CAD, coronary artery disease; aHR, adjusted hazard ratio; CI, confidence interval; ZJU index, Zhejiang University Index; HSI, Hepatic Steatosis Index; FLI, Fatty Liver Index; K-NAFLD score, Korean National Health and Nutrition Examination Survey Non-Alcoholic Fatty Liver Disease Score.

Supplemental Table 10. Area under the curve and optimal cut-offs in participants aged ≥50 years

| **MACE** | | | | | | | | |
| --- | --- | --- | --- | --- | --- | --- | --- | --- |
| Model | AUC of ROC at 10 years (95% CI) | Cut-off value  (95% CI) | Sensitivity  (95% CI) | Specificity  (95% CI) | NPV  (95% CI) | PPV  (95% CI) | NLR  (95% CI) | PLR  (95% CI) |
| ZJU-index | 0.580  (0.565–0.594) | 34.80  (32.99–37.02) | 47.6%  (32.6–63.0) | 64.7%  (49.3–79.4) | 89.5%  (88.7–90.4) | 16.4%  (15.2–19.0) | 0.81  (0.75–0.86) | 1.35  (1.24–1.61) |
| HSI | 0.565  (0.549–0.580) | 32.19  (32.06–34.18) | 55.2%  (41.1–58.5) | 55.4%  (54.2–68.9) | 89.5%  (88.6–90.3) | 15.2%  (14.4–17.0) | 0.81  (0.76–0.87) | 1.24  (1.19–1.39) |
| FLI | 0.592  (0.578–0.606) | 38.55  (21.35–46.16) | 46.4%  (38.8–66.8) | 68.2%  (47.6–75.1) | 89.8%  (89.1–90.8) | 17.4%  (15.4–19.5) | 0.79  (0.70–0.82) | 1.46  (1.27–1.63) |
| K-NAFLD score | 0.596  (0.582–0.611) | -1.75  (-2.47–-0.71) | 52.2%  (38.9–62.3) | 62.9%  (51.4–75.9) | 90.1%  (89.4–90.8) | 16.9%  (15.7–18.7) | 0.76  (0.71–0.81) | 1.41  (1.31–1.57) |
| **CAD** | | | | | | | | |
| Model | AUC of ROC at 10 years (95% CI) | Cut-off value  (95% CI) | Sensitivity  (95% CI) | Specificity  (95% CI) | NPV  (95% CI) | PPV  (95% CI) | NLR  (95% CI) | PLR  (95% CI) |
| ZJU-index | 0.583  (0.564–0.601) | 34.80  (32.78–36.44) | 49.3%  (37.8–66.9) | 63.8%  (46.4–75.2) | 96.0%  (95.6–96.5) | 6.7%  (6.0–7.7) | 0.79  (0.70–0.84) | 1.36  (1.23–1.55) |
| HSI | 0.566  (0.546–0.582) | 32.23  (31.89–34.24) | 56.1%  (42.1–60.4) | 55.0%  (52.2–68.6) | 95.9%  (95.6–96.3) | 6.2%  (5.8–7.1) | 0.80  (0.74–0.86) | 1.25  (1.19–1.40) |
| FLI | 0.593  (0.576–0.609) | 38.56  (19.70–45.06) | 47.2%  (41.3–70.7) | 67.0%  (43.7–72.9) | 96.0%  (95.7–96.7) | 7.1%  (6.0–7.9) | 0.79  (0.65–0.82) | 1.43  (1.23–1.59) |
| K-NAFLD score | 0.595  (0.577–0.595) | -1.75  (-3.23–-0.55) | 52.6%  (38.1–78.9) | 61.7%  (35.4–76.3) | 96.1%  (95.8–97.0) | 6.8%  (5.9–7.9) | 0.77  (0.58–0.82) | 1.37  (1.21–1.61) |
| **Stroke** | | | | | | | | |
| Model | AUC of ROC at 10 years (95% CI) | Cut-off value  (95% CI) | Sensitivity  (95% CI) | Specificity  (95% CI) | NPV  (95% CI) | PPV  (95% CI) | NLR  (95% CI) | PLR  (95% CI) |
| ZJU-index | 0.569  (0.546–0.594) | 32.07  (31.02–39.67) | 71.5%  (22.8–80.6) | 39.3%  (29.6–89.1) | 98.5%  (98.2–98.8) | 2.4%  (2.2–3.7) | 0.72  (0.61–0.89) | 1.18  (1.14–1.85) |
| HSI | 0.556  (0.533–0.582) | 29.13  (28.51–38.93) | 79.3%  (20.1–85.1) | 29.7%  (24.7–88.5) | 98.6%  (98.1–98.8) | 2.3%  (2.1–3.7) | 0.70  (0.58–0.91) | 1.13  (1.10–1.88) |
| FLI | 0.585  (0.563–0.609) | 14.08  (11.74–43.87) | 79.1%  (42.2–83.5) | 33.7%  (29.1–71.4) | 98.7%  (98.3–98.9) | 2.4%  (2.2–3.2) | 0.62  (0.53–0.82) | 1.19  (1.17–1.54) |
| K-NAFLD score | 0.586  (0.563–0.611) | -2.13  (-3.67–-1.73) | 59.2%  (51.6–87.1) | 55.3%  (26.5–61.7) | 98.5%  (98.3–99.0) | 2.7%  (2.3–3.1) | 0.74  (0.49–0.80) | 1.33  (1.17–1.45) |

Time-dependent receiver operating characteristic (ROC) analyses were conducted using univariable Cox models including each hepatic steatosis index. The area under the ROC curve (AUC) and corresponding 95% CIs were calculated for 10-year follow-up. Optimal cut-off values were determined using the Youden index, with corresponding sensitivity, specificity, negative predictive value (NPV), positive predictive value (PPV), negative likelihood ratio (NLR), and positive likelihood ratio (PLR). Abbreviations: MACE, major adverse cardiovascular events; CAD, coronary artery disease; AUC, area under the ROC curve; NPV, negative predictive value; PPV, positive predictive value; NLR, negative likelihood ratio; PLR, positive likelihood ratio; ZJU index, Zhejiang University Index; HSI, Hepatic Steatosis Index; FLI, Fatty Liver Index; K-NAFLD score, Korean National Health and Nutrition Examination Survey Non-Alcoholic Fatty Liver Disease Score.

Supplemental Table 11. Pairwise comparisons of AUCs for predicting cardiovascular outcomes in participants aged ≥50 years

| MACE |  |  |  |
| --- | --- | --- | --- |
| Comparison | ΔAUC | 95% CI | P value |
| HSI - ZJU index | -0.015 | -0.020, -0.009 | <0.001 |
| FLI - ZJU index | 0.012 | 0.004, 0.020 | 0.004 |
| K-NAFLD score - ZJU index | 0.017 | 0.009, 0.025 | <0.001 |
| FLI - HSI | 0.027 | 0.016, 0.037 | <0.001 |
| K-NAFLD score - HSI | 0.031 | -0.021, -0.040 | <0.001 |
| K-NAFLD score - FLI | -0.004 | -0.003, 0.004 | 0.266 |
| CAD |  |  |  |
| Comparison | ΔAUC | 95% CI | P value |
| HSI - ZJU index | -0.018 | -0.025, -0.011 | <0.001 |
| FLI - ZJU index | 0.009 | -0.001, 0.019 | 0.064 |
| K-NAFLD score - ZJU index | 0.012 | 0.002, 0.022 | 0.02 |
| FLI - HSI | 0.027 | 0.014, 0.041 | <0.001 |
| K-NAFLD score - HSI | 0.029 | 0.017, 0.041 | <0.001 |
| K-NAFLD score - FLI | -0.002 | -0.007, 0.012 | 0.642 |
| Stroke |  |  |  |
| Comparison | ΔAUC | 95% CI | P value |
| HSI - ZJU index | -0.013 | -0.023, -0.005 | 0.006 |
| FLI - ZJU index | 0.016 | 0.001, 0.032 | 0.046 |
| K-NAFLD score - ZJU index | 0.017 | 0.003, 0.030 | 0.010 |
| FLI - HSI | 0.029 | 0.011, 0.050 | 0.006 |
| K-NAFLD score - HSI | 0.030 | 0.013, 0.047 | <0.001 |
| K-NAFLD score - FLI | 0.001 | -0.012, 0.013 | 0.982 |

Pairwise differences in AUCs between hepatic steatosis indices were calculated using 1,000 bootstrap resamples to determine 95% CIs and p-values. Abbreviations: MACE, major adverse cardiovascular events; CAD, coronary artery disease; AUC, area under the ROC curve; CI, confidence interval; ZJU index, Zhejiang University Index; HSI, Hepatic Steatosis Index; FLI, Fatty Liver Index; K-NAFLD score, Korean National Health and Nutrition Examination Survey Non-Alcoholic Fatty Liver Disease Score.

Supplemental Table 12. Area under the curve and optimal cut-offs for predicting cardiovascular outcomes in participants with a high cardiovascular risk profile

| MACE | | | | | | | | |
| --- | --- | --- | --- | --- | --- | --- | --- | --- |
| Model | AUC of ROC at 10 years (95% CI) | Cut-off value  (95% CI) | Sensitivity  (95% CI) | Specificity  (95% CI) | NPV  (95% CI) | PPV  (95% CI) | NLR  (95% CI) | PLR  (95% CI) |
| ZJU-index | 0.53  (0.51–0.54) | 39.65  (36.70–40.78) | 27.1%  (21.1–48.5) | 78.5%  (57.3–83.8) | 94.1%  (93.8–94.4) | 7.9%  (7.0–8.6) | 0.93  (0.89–0.95) | 1.26  (1.12–1.38) |
| HSI | 0.51  (0.50–0.52) | 38.98  (32.12–43.32) | 26.3%  (11.1–71.4) | 76.1%  (31.3–90.9) | 93.8%  (93.6–94.3) | 6.9%  (6.5–8.0) | 0.97  (0.91–0.98) | 1.10  (1.03–1.28) |
| FLI | 0.54  (0.53–0.56) | 44.65  (33.25–50.09) | 54.2%  (47.3–67.2) | 53.2%  (39.4–59.5) | 94.5%  (94.1–94.9) | 7.3%  (6.9–7.7) | 0.86  (0.81–0.90) | 1.16  (1.11–1.22) |
| K-NAFLD score | 0.54  (0.53–0.55) | -1.61  (-1.75–-0.26) | 64.9%  (43.5–68.1) | 42.2%  (39.6–63.3) | 94.7%  (94.2–95.0) | 7.1%  (6.8–7.8) | 0.83  (0.78–0.90) | 1.12  (1.10–1.23) |
| CAD | | | | | | | | |
| Model | AUC of ROC at 10 years (95% CI) | Cut-off value  (95% CI) | Sensitivity  (95% CI) | Specificity  (95% CI) | NPV  (95% CI) | PPV  (95% CI) | NLR  (95% CI) | PLR  (95% CI) |
| ZJU-index | 0.53  (0.52–0.55) | 39.65  (36.02–40.09) | 27.7%  (24.6–55.4) | 78.4%  (50.9–80.6) | 95.8%  (95.7–96.2) | 5.7%  (4.9–6.2) | 0.92  (0.85–0.94) | 1.28  (1.11–1.41) |
| HSI | 0.52  (0.50–0.53) | 34.74  (32.04–40.39) | 53.6%  (20.9–73.1) | 49.9%  (30.5–82.2) | 95.8%  (95.5–96.1) | 4.8%  (4.6–5.5) | 0.93  (0.87–0.97) | 1.07  (1.04–1.23) |
| FLI | 0.55  (0.53–0.56) | 44.68  (37.73–48.68) | 55.5%  (50.2–63.3) | 53.2%  (44.9–58.0) | 96.2%  (96.0–96.5) | 5.3%  (5.0–5.6) | 0.84  (0.78–0.88) | 1.18  (1.13–1.25) |
| K-NAFLD score | 0.55  (0.54–0.56) | -0.80  (-1.64–-0.35) | 53.3%  (46.7–68.8) | 55.7%  (41.6–62.1) | 96.2%  (96.0–96.6) | 5.4%  (5.0–5.8) | 0.84  (0.75–0.87) | 1.20  (1.13–1.28) |
| Stroke | | | | | | | | |
| Model | AUC of ROC at 10 years (95% CI) | Cut-off value  (95% CI) | Sensitivity  (95% CI) | Specificity  (95% CI) | NPV  (95% CI) | PPV  (95% CI) | NLR  (95% CI) | PLR  (95% CI) |
| ZJU-index | 0.52  (0.50–0.54) | 40.61  (35.31–43.57) | 22.2%  (12.0–62.2) | 82.9%  (43.8–92.5) | 98.1%  (98.0–98.3) | 2.6%  (2.1–3.4) | 0.94  (0.87–0.96) | 1.30  (1.08–1.75) |
| HSI | 0.50  (0.48–0.52) | 42.85  (28.64–50.49) | 12.2%  (3.0–90.9) | 89.8%  (11.6–98.9) | 98.1%  (98.0–98.5) | 2.4%  (1.9–4.4) | 0.98  (0.75–0.99) | 1.20  (1.02–2.34) |
| FLI | 0.53  (0.51–0.55) | 32.29  (13.69–76.45) | 67.3%  (19.0–90.9) | 37.8%  (13.3–85.4) | 98.3%  (98.0–98.7) | 2.1%  (2.0–2.8) | 0.87  (0.68–0.95) | 1.08  (1.05–1.44) |
| K-NAFLD score | 0.53  (0.51–0.55) | 1.44  (-3.42–1.78) | 60.4%  (24.3–91.8) | 44.7%  (12.4–81.6) | 98.2%  (98.1–98.8) | 2.2%  (2.0–2.6) | 0.89  (0.62–0.95) | 1.09  (1.04–1.31) |

Time-dependent receiver operating characteristic (ROC) analyses were conducted using univariable Cox models including each hepatic steatosis index. The area under the ROC curve (AUC) and corresponding 95% CIs were calculated for 10-year follow-up. Optimal cut-off values were determined using the Youden index, with corresponding sensitivity, specificity, negative predictive value (NPV), positive predictive value (PPV), negative likelihood ratio (NLR), and positive likelihood ratio (PLR). Abbreviations: MACE, major adverse cardiovascular events; CAD, coronary artery disease; AUC, area under the ROC curve; NPV, negative predictive value; PPV, positive predictive value; NLR, negative likelihood ratio; PLR, positive likelihood ratio; ZJU index, Zhejiang University Index; HSI, Hepatic Steatosis Index; FLI, Fatty Liver Index; K-NAFLD score, Korean National Health and Nutrition Examination Survey Non-Alcoholic Fatty Liver Disease Score.

Supplemental Table 13. Pairwise comparisons of AUCs for predicting cardiovascular outcomes in the high cardiovascular risk subgroup

| MACE |  |  |  |
| --- | --- | --- | --- |
| Comparison | ΔAUC | 95% CI | P value |
| HSI - ZJU index | -0.017 | -0.022–-0.012 | <0.001 |
| FLI - ZJU index | 0.016 | 0.009–0.024 | <0.001 |
| K-NAFLD score - ZJU index | 0.015 | 0.008–0.023 | <0.001 |
| FLI - HSI | 0.033 | 0.024–0.043 | <0.001 |
| K-NAFLD score - HSI | 0.032 | 0.024–0.041 | <0.001 |
| K-NAFLD score - FLI | -0.001 | -0.009–0.007 | 0.756 |
| CAD |  |  |  |
| Comparison | ΔAUC | 95% CI | P value |
| HSI - ZJU index | -0.017 | -0.023–-0.011 | <0.001 |
| FLI - ZJU index | 0.014 | 0.006–0.022 | <0.001 |
| K-NAFLD score - ZJU index | 0.017 | 0.008–0.025 | <0.001 |
| FLI - HSI | 0.031 | 0.021–0.042 | <0.001 |
| K-NAFLD score - HSI | 0.034 | 0.024–0.043 | <0.001 |
| K-NAFLD score - FLI | 0.002 | -0.007–0.012 | 0.634 |
| Stroke |  |  |  |
| Comparison | ΔAUC | 95% CI | P value |
| HSI - ZJU index | -0.016 | -0.024–-0.008 | <0.001 |
| FLI - ZJU index | 0.011 | -0.002–0.025 | 0.108 |
| K-NAFLD score - ZJU index | 0.011 | -0.002–0.024 | 0.098 |
| FLI - HSI | 0.027 | 0.012–0.044 | 0.002 |
| K-NAFLD score - HSI | 0.026 | 0.012–0.041 | <0.001 |
| K-NAFLD score - FLI | -0.001 | -0.015–0.013 | 0.982 |

Pairwise comparisons of AUCs between hepatic steatosis indices were performed using 1,000 bootstrap resampling iterations to estimate 95% confidence intervals (CIs) and P values. Abbreviations: MACE, major adverse cardiovascular events; CAD, coronary artery disease; AUC, area under the receiver operating characteristic curve; CI, confidence interval; ZJU index, Zhejiang University Index; HSI, Hepatic Steatosis Index; FLI, Fatty Liver Index; K-NAFLD score, Korean National Health and Nutrition Examination Survey Non-Alcoholic Fatty Liver Disease Score.

Supplemental Table 14. Baseline Characteristics of Included Participants and Those Excluded from the Analysis

|  | Included | Excluded | p.value |
| --- | --- | --- | --- |
| Age(y) | 45.0 (40.0, 50.0) [n=134604] | 49.0 (43.0, 57.0) [n=19885] | <0.001 |
| Men, n (%) | 102227 (75.9%) [n=134604] | 11840 (59.5%) [n=19885] | <0.001 |
| BMI(kg/m²) | 22.9 (20.9, 25.2) [n=134604] | 22.6 (20.4, 25.0) [n=19840] | <0.001 |
| Height(cm) | 169.0 (163.1, 173.7) [n=134604] | 165.4 (159.2, 171.1) [n=19840] | <0.001 |
| WC(cm) | 82.0 (75.9, 88.0) [n=134604] | 81.1 (74.3, 88.0) [n=19614] | <0.001 |
| Systolic blood pressure(mmHg) | 119.0 (109.0, 128.0) [n=134604] | 119.0 (108.0, 129.0) [n=19843] | 0.051 |
| Diastolic blood pressure(mmHg) | 74.0 (67.0, 82.0) [n=134604] | 74.0 (66.0, 82.0) [n=19843] | <0.001 |
| Fasting plasma glucose(mg/dL) | 92.0 (87.0, 99.0) [n=134604] | 93.0 (86.0, 101.0) [n=19439] | <0.001 |
| Tryglycerides(mg/dL) | 91.0 (63.0, 136.0) [n=134604] | 86.0 (60.0, 128.0) [n=19662] | <0.001 |
| HDL cholesterol(mg/dL) | 58.0 (49.0, 69.0) [n=134604] | 61.0 (51.0, 73.0) [n=19679] | <0.001 |
| LDL cholesterol(mg/dL) | 123.0 (103.0, 145.0) [n=134604] | 122.0 (101.0, 144.0) [n=19662] | <0.001 |
| AST(IU/L) | 20.0 (17.0, 25.0) [n=134604] | 20.0 (17.0, 25.0) [n=19693] | <0.001 |
| ALT(IU/L) | 20.0 (14.0, 30.0) [n=134604] | 18.0 (13.0, 26.0) [n=19693] | <0.001 |
| GGT(IU/L) | 28.0 (19.0, 48.0) [n=134604] | 25.0 (17.0, 44.0) [n=19690] | <0.001 |
| smoking(none/past/current),n,(%) | 70205/24247/40152 (52.2/18.0/29.8) [n=134604] | 10510/3304/5791 (53.6/16.9/29.5) [n=19605] | <0.001 |
| Alcohol consumption,n,(+)(%) | 28117 (20.9%) [n=134604] | 3861 (19.9%) [n=19394] | 0.002 |
| Physical exercise,n,(+)(%) | 24655 (18.3%) [n=134604] | 3841 (19.6%) [n=19563] | <0.001 |
| Diabetes medication use, n, (+)(%) | 3239 (2.4%) [n=134604] | 780 (4.0%) [n=19642] | <0.001 |
| Antihypertensive medication use, n, (+)(%) | 10925 (8.1%) [n=134604] | 2353 (12.0%) [n=19645] | <0.001 |
| Dyslipidemia medication use, n, (+)(%) | 5513 (4.1%) [n=134604] | 1136 (5.8%) [n=19642] | <0.001 |

For categorical variables, n (%) is presented. For continuous variables, median (interquartile range) is presented. Alcohol consumption (+) indicates alcohol intake above the predefined threshold (≥20 g/day for women and ≥30 g/day for men). P values were calculated for comparisons between included and excluded participants. Because of missing values, the number of participants contributing to each variable may differ, and the corresponding sample size is shown in brackets for each characteristic. Excluded participants include individuals excluded due to missing data or loss to follow-up.

Abbreviations: BMI, body mass index; WC, waist circumference; LDL, low-density lipoprotein; HDL, high-density lipoprotein.
