# Supplementary figures and images for: Steatotic liver disease indices for cardiovascular event prediction: Panasonic cohort study 28
Source: Am J Prev Cardiol. 2026 Mar 10;27:101541. doi: 10.1016/j.ajpc.2026.101541 (PMC13261237; doi:10.1016/j.ajpc.2026.101541)

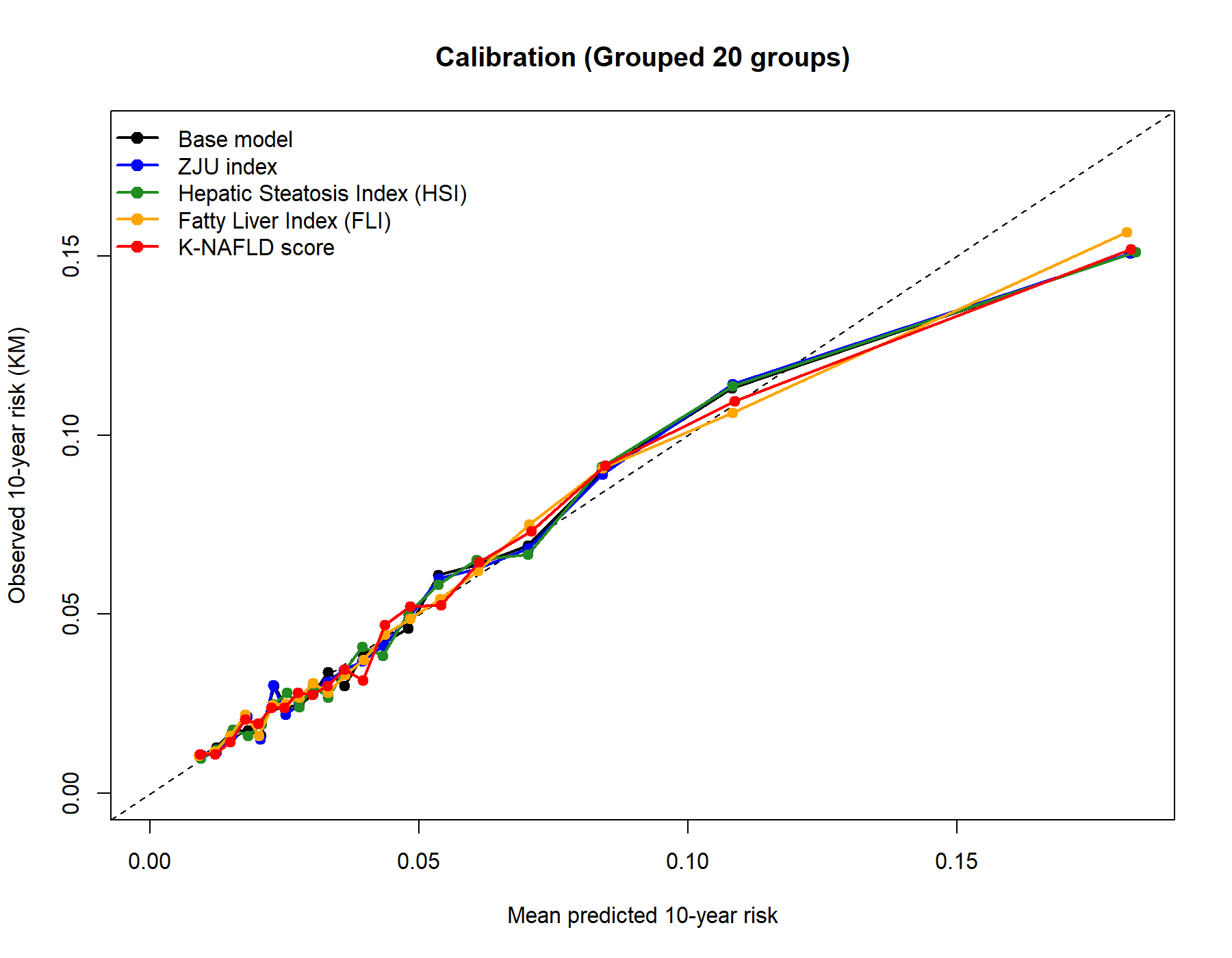

Supplement: Supplementary file 2 [file mmc2.zip › Supplemental Figure 1.png]
